# Solidarity in a Community of Nursing Colleagues

**DOI:** 10.1177/23779608211009514

**Published:** 2021-04-12

**Authors:** Margareth Kristoffersen

**Affiliations:** Department of Care and Ethics, Faculty of Health Sciences, University of Stavanger, Stavanger, Norway

**Keywords:** community of nursing colleagues, destructive behavior, secondary analysis, solidarity, support, sympathy

## Abstract

**Introduction:**

Several concepts have been used to describe the qualities of communities of nursing colleagues. Nonetheless, few studies have shed light on nursing communities by drawing on the concept of solidarity.

**Objective:**

To explore solidarity among a community of nursing colleagues.

**Methods:**

A qualitative research design with a reflective life world approach was selected. This study reused data from a larger Norwegian empirical study. The data from the original study consisted of qualitative interviews and follow-up interviews with 13 nurses (RNs). The research context was municipality and specialist health services. A secondary data analysis was conducted. The study was based on the SRQR reporting guidelines.

**Results:**

The results were formulated under two themes: 1) having indispensable relationships and 2) encountering a relative absence of sympathy.

**Conclusion:**

A sense of community among nursing colleagues seems to rely on solidarity: whatever affects one nurse affects another. The solidarity that arose from the content of commonalities involved maintaining indispensable relationships with nursing colleagues by supporting and aiding them and simultaneously enduring a relative absence of sympathy. Solidarity among the community in this study was not a peripheral concept of the general notion of solidarity, implying that the commonalities within the collegial relationships were ambiguous and could shift from something good to something relatively good and vice versa. Such a shift was evidenced by nurses’ experiences of their community.

## Introduction

Extensive attention has been dedicated to describing the qualities of communities of nursing colleagues. Several concepts have been used, such as work group cohesion, group support, coworker support, intraprofessional relationships, and peer relationships (Duddle & Boughton, 2007; Topa & Moriano, 2013). A community can appear in any area and is formed through earnest activity intended to do or accomplish something over time (Wenger, 1998). Communities of nursing colleagues influence individual nurses, i.e., registered nurses (RNs) with the same social standing in their organizations (Purpora et al., 2014). In a dialogue within the nursing discipline, communities develop professional conduct, help to ensure nurses’ professional conduct and enable them to accomplish a shared goal of high-quality, effective nursing practice (Benner, 2000; Benner et al., 2010). Furthermore, professional conduct incorporates complex processes of “embodiment, emotion and lifeworld for rationality and agency in nursing practice” (Benner, 2000, p. 5), in which taking charge is of ultimate concern (Havig et al., 2013; World Health Organization, 2014, 2019). However, without communities of nursing colleagues, developing embodied professional knowledge would have been impossible for the individual (Benner, 2000). Communities are based on core nursing competencies (Institute of Quality and Safety Education for Nurses, 2003). Collaboration between colleagues constitutes one of six competencies. The other five competencies are patient-centered care, evidence-based practice, quality improvement, safety and informatics. Communities represent a collection of forces that affect nurses’ desires to stay in their current position and are an indicator of the long-term feasibility of jobs (Gilmartin, 2012; Liebermann et al., 2015; Kristoffersen, 2021).

Supportive actions and behaviors are qualities within communities that are considered a prerequisite to prevent inadequate nursing care (Zuniga et al., 2015). Support involves openly discussing patient care concerns and being comfortable asking colleagues for assistance in caring for patients when needed (Elenbecker, 2006). Nurses look to colleagues for support to address challenges (Bjarnadottir, 2011); in particular, colleagues with a good influence are often involved in collaboration. Moreover, supportive relationships represent a job satisfier (Purpora & Blegen, 2015). Importantly, an apparent association between supportive relationships and reduced work stressors has been recognized (Zuniga et al., 2015). A reduction in work stressors can thus lead to positive effects on nurses’ health (Bjarnadottir, 2011; Halbesleben & Wheeler 2008; Llorens et al., 2007).

Less supportive actions and behaviors also seem to be qualities within communities of nursing colleagues. Research has documented that horizontal violence is considered a pervasive problem (Becher & Visovsky, 2012; Budin et al., 2013; Demir & Rodwell, 2012; Purpora & Blegen, 2015; Topa & Moriano, 2013), and verbal abuse is often the most common form of violence experienced by nurses (Farrell et al., 2006). Such actions and behavior can be repetitive (Topa & Moriano, 2013; Vessey et al., 2010) and are characterized by situations occurring over a period of time that are carried out by one or several colleagues, while the bullied individual experiences difficulties defending himself or herself (Demir & Rodwell, 2012; Rodwell & Demir, 2012). When destructive behavior increases, relationships become less supportive (Duddle & Boughton, 2007; Purpora & Blegen, 2015; Purpora et al., 2014; Topa & Moriano, 2013). This process often involves a lack of trust among colleagues and difficulty asking for necessary assistance. In addition, individual nurses’ moods are affected (Elenbecker, 2006), and they may feel lonely, vulnerable and humiliated or abused and frustrated (Demerouti, et al., 2000; O’Connell et al., 2000; Topa & Moriano, 2013; Vessey, et al., 2010). Moreover, bullied nurses may feel incompetent and incapable, leading to a greater risk of errors occurring in nursing practice (Purpora et al., 2014; Wilson, 2016).

The concept of solidarity may provide an understanding of nurses’ experiences in relation to their colleagues. This necessitates an exploration of solidarity among nursing colleagues. The research that focuses on solidarity ranges from relationships at the microlevel to social contexts at the macrolevel (Itzkovich & Heilbrunn, 2016; Prainsack & Buyx, 2018). This study draws on the Danish philosopher and theologian Knud E. Løgstrup’s (1993) description of solidarity to provide a human and universal or moral-philosophical perspective on the concept.

## Background

Solidarity comes from the term “solidum” [lat.], which means the whole (Kortner et al., 1980, p. 648) that is unreduced and unclipped (Løgstrup, 1993). Regarding the unreduced nature of solidarity, Løgstrup (1993) bases the concept of solidarity on the assumption of relationality as a given life condition according to which human beings are mutually dependent on one another. He perceives relationality as an integral part of solidarity. Solidarity is associated with mutual relationships that include taking care of each other’s best interests and whatever else in the other person’s life depends upon us. In other words, solidarity becomes the “soil” for love (Løgstrup, 1993, p.21). However, relationality is not solidarity. Løgstrup (1993) notes that two aspects must be added to characterize solidarity: “what is common and the obligation that arises from the common” (p. 8).

“What is common” or the content of what is common can be anything (Løgstrup, 1993), implying that what is common is open and must be understood in context. Developing connection via human relationships or among a particular group of people makes “what is common” more ambiguous than being connected to the good. Løgstrup (1993) states that solidarity becomes as ethically ambiguous as the content of the common. As the content of what is shared stands open, solidarity does not protect against the relatively good or the less good. In cases in which the common is less good within a community, i.e., the shared content confronts or conflicts with ethical actions and behaviors, sympathy may be lacking. This lack often occurs when tensions exist and something evokes uncertainty or reactions such as irritation, dissatisfaction, or antipathy (Løgstrup, 1991).

The aspect of “obligation” that arises from “what is common” emphasizes that the content of what is common gives rise to an effort: “what to do or how to do it” (Løgstrup, 1993, p. 7). Therefore, solidarity can be understood as an implication, but the obligation does not need to be explicitly defined. Nonetheless, the obligation involves supporting the other based on what is common and standing together simply because something is common (Løgstrup, 1993). The emphasis is on the debt to others, i.e., being indebted to someone or something, such as a moral debt of gratitude for received help that is owed by one person. Although the aspects of “what is common” and “the obligation that arises from the common” are open to interpretation, Løgstrup (1993) underscores that solidarity is never so openly defined that it would no longer be ethical. Furthermore, solidarity can be bad when exploitation occurs and one derives profit due to the exploitation; however, an important criterion of solidarity is that one aims to overcome the bad or obtain the good in life (Løgstrup, 1991).

In an American study, Benner (2000) refers to Løgstrup (1991) and relates solidarity to “the pre-ethical, precultural moral sources, as embodied in our human responses to the other” (p. 10), i.e., “life manifestations of trust, mercy, openness of speech” (p. 14) making it possible to take care of the other. Schwartz and Abbott (2007) found that storytelling promoted nursing students’ solidarity with patients. Consequently, nurses’ capacity of listening to patients’ stories created feelings of unity based on a common interest. In a Turkish study, Uslusoy and Alpar (2013) describe the concept of solidarity as an attitude including three dimensions: emotional solidarity, academic solidarity and negative opinions about solidarity. They state that solidarity represents support between colleagues and the sharing of professional knowledge and skills with each other. Moreover, solidarity may contribute to the development of colleagues’ competence and increase the quality of nursing practice by enabling efficient collaboration and protecting colleagues from destructive or undesirable behaviors. Previous research has also found that solidarity between colleagues in the long-term care sector contributes to success in the organization (Cramm et al., 2013). In an Israeli study of 15 organizations, Itzkovich and Heilbrunn (2016) demonstrated that an increased level of solidarity was associated with lower levels of incivility. Nevertheless, little documentation is available on how solidarity is expressed among nurses and how the aspects of “what is common” and “the obligation that arises from what is common” (Løgstrup, 1993) influence individual nurses, reflecting a need for a more in-depth understanding of how solidarity can shed light on nurses’ experiences within their communities. Furthermore, solidarity in a community of nursing colleagues can be ambiguous rather than being connected to the good. The good and the less good can be intertwined. A similar observation was indicated in a previous study (Kristoffersen, 2019a). Thus, the aim of this study was to explore solidarity among a community of nursing colleagues.

## Method

### Design

A qualitative research design was selected (Polit & Beck, 2018) with “a reflective life world” approach (Lindberg et al., 2016, p. 3) to contribute to insight into the participants’ experiences. The approach was based on methodological principles such as openness and flexibility in adopting a reflective attitude and avoiding haste in an attempt to “make definite what is indefinite” (Lindberg et al., 2016, p. 3). This study was a secondary analysis (Heaton, 2004). The data were derived from a previous, larger Norwegian empirical study with a hermeneutical research design that aimed to identify what is important for nurses to remain in daily nursing practice (Kristoffersen, 2013). The empirical data in the study motivated a secondary analysis to shed light on the data and the associated implications in relation to solidarity in a community of nursing colleagues. Gaining a more in-depth understanding of the empirical data emerged as a matter of interest (Heaton, 2004), and drawing on philosophy to further understand the data was considered a valuable approach (Lindberg et al., 2016). Løgstrup’s (1993) clarification of solidarity was used as an analytical tool to explore the aim of the study. The study was based on the Standard for Reporting Qualitative Research (SRQR) guidelines (O’Brien et al., 2014).

### Sample and Participants

The larger original study (Kristoffersen, 2013) employed a nonprobability sampling method with purposive sampling procedures (Polit & Beck, 2018). The participant selection criteria were a minimum of two years of nursing experience and full-time or almost full-time work within acute and long-term physical or mental municipalities and specialized health services. Eligible participants were recruited by their formal nursing leader. To ensure that the participants would reflect on and articulate their perspectives on the study’s aim, they were asked to contact the researcher directly.

Thirteen RNs (11 women and 2 men) agreed to participate, 12 of whom had advanced education. The participants had varying work experience ranging from two to forty years and had full-time or almost full-time employment as physical or mental health nurses (i.e., within nursing homes, home-based care and mental, intensive, medical and surgical units). Many of them had worked for ten years or more on the same ward. Their ages were between 26 and 62 years (median 51 years). The researcher had no relationship with the participants.

### Data Collection

The data were collected by rereading the interview transcripts from the larger original study to identify the most suitable parts of the empirical material to understand how nurses expressed their experiences of their relationships with their nursing colleagues and to comprehensively understand these expressions (see Heaton, 2004). The larger original study consisted of 27 qualitative interviews and qualitative follow-up interviews (Kristoffersen, 2013). The use of follow-up interviews was intended to allow time for reflection after the first interview to elicit more in-depth and broader information regarding the participants’ perceptions of their day-to-day experiences of caring for patients (see Kvale & Brinkman, 2009; Silverman, 2006). In the interviews, the participants were asked to elaborate freely and at length on what was important for them to remain in daily nursing practice. To encourage the participants to describe their experiences further, they were asked to elaborate on what they already had described. Specific questions were also posed, such as, “What can you tell me about what is important to you each day when you go to work?” and “What gives you pleasure in daily nursing practice?”

### Ethical Considerations

The data were drawn from the original empirical study approved by the Norwegian Center for Research Data. Formal nursing leaders provided information about the study to the participants. The researcher obtained consent from the participants before the interview started. The data were limited and anonymized. Details that might contribute to the identification of the participants were omitted in the description of the participants and the presentation of the results.

### Analysis

Based on principles of methodological support to capture the information in the empirical data (Lindberg et al., 2016), the first step of the secondary data analysis involved reading and rereading the data to determine a general structure of the empirical material and identify new patterns of meanings. In the next step, a philosophical examination was performed whereby the philosophical texts and the data were read again (Lindberg et al., 2016). The reading involved analysis through exploration with an open attitude to obtain an understanding of the data in relation to solidarity, which required a process of deep reflection. The analytical strategy involved posing several reflective questions about the material, focusing on what the material said, what it meant or revolved around and what it implied. For example, the questions asked what the data indicated about solidarity, what was common within the community of nursing colleagues, what obligations arose from what was common, and how aspects of solidarity could shed light on the day-to-day experiences of nursing practice. In addition, in the data, what appeared to be obvious and more latent meaning structures were considered. In this way, a more in-depth understanding of the data emerged (Lindberg et al., 2016), resulting in the description of themes (see Table 1, Examples from the analysis process).

**Table 1.** Examples From the Analysis Process.

Examples from the empirical data	General structure	Meaning structure	Viewed against the philosophy
*A good relationship with colleagues is very important; as long as I thrive among colleagues, I feel like I can do almost anything.* *Good relationships with colleagues promote things to go very well. In turn, I think we thrive when working together and are a fine team. When the work flows easily, it involves recognition of our relationships, both the colleagues and me.* *I think collegial relationships must be a basis for going to the patients and coming back to colleagues when having experienced tough things and feeling powerless.* *Many nurses are doing a fantastic job. They catch important things related to the patient and ‘fix it’. This means that they dare to stand up for the patient with professionality. I would not be afraid to let them take care of any of my loved ones. My colleagues have high professional awareness and discuss relevant issues that I’m interested in and need to discuss. It is a good thing. It makes me aware and proud of the performance of quality nursing care.* *We had a kind of “I’m agreeing with you attitude”, and we thematize issues by asking, “What is the problem? Can we manage the problem, or do we need assistance from other professionals?”* *Colleagues do understand what I’m talking about, and thus, when the situation is tough and tragic things happen, it is the colleagues who are staying there. We stay together within the situation.* *When tragic things have happened with a patient, then it has been possible to come through with the help of colleagues.* *When we get strong impressions in meeting with patients, it revolves around ‘working in teams’, and we are mutually dependent on each other as colleagues and need to stay together.* *Having experienced challenging situations and managed them, it can nonetheless be a tough thing going back to work. However, I often came through such situations with the help of colleagues. We stay together. It gives me a feeling of being stronger than I was before.*	Nurses would not have given up the content of their relationships with nursing colleagues for anything in the world. The relationships gave a sense of being cared about and provided a sympathetic atmosphere. Support was a common experience. The nurses verbally expressed how much they appreciated seeing each other, which was important at the beginning of a working day and created a basis for a good day. Colleagues were perceived as great and talented colleagues who deserved to be honored. Colleagues offered support by explicitly telling each other how professionally inspired they felt from working together. Staying together was crucial for nurses, particularly when unforeseen situations occurred and nurses had to rely on each other to accomplish difficult tasks.	Having indispensable relationships	Solidarity is based on the assumption of relationality as a given life condition according to which human beings are mutually dependent on one another and taking care of each other’s best interests (Løgstrup, 1993). Relationality is perceived as an integral part of solidarity (Løgstrup, 1993). However, two central aspects must be added to characterize solidarity: what is common and the obligation that arises from the common (Løgstrup, 1993, p. 8). The obligation involves supporting the other based on what is common and standing together simply because something is common (Løgstrup, 1993).
*It is rather hard when you have a sense of being rejected or ignored and nobody cares.* *Nursing practice is often performed ‘in the middle of a cauldron’.* *Some colleagues can contribute to a different work atmosphere, and we have to struggle with them. In addition, the patients are very ill. In a way, the working days become doubly hard, and going to work becomes challenging when our collegial relationships are difficult.* *Colleagues can be unable to adapt, and when you face situations where colleagues really should not have been there, it is tough and inhibits me and the work I have to do. It’s not easy to get rid of that colleague either.* *Experiencing that colleagues did not welcome me or didn’t like me coming to the job represents a barrier to go to work.* *Struggling with colleagues represents a double struggle.* *I have experienced negative colleagues as an inhibitor.* *I’m doing complex and very uncomfortable work, and despite this, I don’t get the help that I need from colleagues when caring for very ill patients.* *The times I most dread going to work is when there has been a collegial conflict. This is one of the most tiring things that exists; it drains your energy.* *Experiencing that colleagues did not welcoming me or didn’t like me coming to the job represents a barrier to go to work.*	The content of what was common among colleagues involved a relative absence of sympathy that gave rise to effort: maintaining indispensable relationships and demonstrating commitment to the community of nursing colleagues became demanding. Negative verbal or nonverbal responses influenced individual nurses’ destinies, causing difficulty in understanding what to do and how to do it when wanting to look out for colleagues’ best interests. A relative absence of sympathy caused thoughts and feelings described as resentment and recrimination and thus a loss of the desire to work	Encountering a relative absence of sympathy	Solidarity is based on the assumption of relationality as a given life condition (Løgstrup, 1993). The content of what is common can be anything, implying that what is common stands open and must be understood in context (Løgstrup, 1993). The obligation involves standing together simply because something is common (Løgstrup, 1993). A lack of sympathy often occurs when tensions exit and something evokes uncertainty or reactions such as irritation, unsatisfaction, or antipathy (Løgstrup, 1991).

## Results

Two themes emerged from the analysis: 1) having indispensable relationships and 2) encountering a relative absence of sympathy.

### Having Indispensable Relationships

Nurses’ common perception of having indispensable relationships suggests that the nurses would not give up the content of their relationships with their nursing colleagues for anything in the world. These relationships gave nurses a sense of being dependent on one another and provided a sympathetic atmosphere in which the nurses felt comfortable. Thus, the content of commonalities gave rise to an obligation to take care of or support each other as colleagues. The data show that support was a common experience. Support included verbal expressions that were considered to be sympathetic since they contributed to the nurses’ energy and promoted involvement in a community. The colleagues supported one another by kindly expressing how much they appreciated seeing each other, which was important at the beginning of a working day and created the foundation for a good day. The nurses stated that it had always been that way and would be forever. One nurse said, “A good relationship with colleagues is very important; as long as I thrive among colleagues, I feel like I can do almost anything”.

In their descriptions of their colleagues’ nursing practice, the nurses described more support than what would generally be expected. Their descriptions included comments that nurses were great and talented colleagues who deserved to be honored because they cared for patients in a professional manner and understood how sickness and suffering often changed a person. One nurse said,

Many nurses are doing a fantastic job. They catch important things related to the patient and “fix it”. This means that they dare to stand up for the patient with professionality. I would not be afraid to let them take care of any of my loved ones.

Colleagues offered support by explicitly telling each other that they were professionally inspired by working together. Positive feedback related to the performance of nursing practice contributed to a sense of being connected to the community; the community therefore functioned as a social and cultural resource that increased learning and understanding among the nurses. However, the nurses also evaluated the quality of the support and differentiated between good and less good verbal support. One nurse said, “Sometimes praise is like extravagant praise, meaning a bit easy, and such praise does not matter as much as genuine praise”. A sincerely spoken word or short sentence was valuable and could be sufficient to generate a sense of being a skilled professional, while many empty words were not. Relationships with colleagues were indispensable as they provided opportunities to vent thoughts and feelings. Unresolved feelings and issues could be addressed and were allowed to be voiced. Such support contributed to a sense of being on the same wavelength with each other. Thus, the nurses had someone to turn to when they confronted complex challenges and had to explore options. The nurses stated that they needed to know who could provide them with support and share experiences of painful or difficult issues.

Staying together was crucial, particularly when unforeseen situations occurred and nurses had to rely on each other to accomplish difficult tasks. The nurses felt dependent on colleagues who could be trusted and were capable of providing help to patients. These colleagues provided a safe environment by working in an appropriate way to collaborate within a community. Colleagues’ actions and behaviors were experienced as appropriate when these actions enhanced a good team approach to caring for patients. A nurse said, “When we get strong impressions in meeting with patients, it revolves around ‘working in teams’, and we are mutually dependent on each other as colleagues and need to stay together”. Colleagues were considered to be best qualified to provide aid. Their aid was based on embodied knowledge that was learned in the same or similar nursing situations. Another nurse said,

Colleagues do understand what I’m talking about, and thus, when the situation is tough and tragic things happen, it is the colleagues who are staying there. We stay together within the situation. No one else understands. Understanding necessitates having been there. Having been there when the situation happened provides embodied knowledge.

In contrast, continuing to work in a community without enriching and sympathetic nursing colleagues who were willing to support and aid each other would have been considered difficult. One nurse said the following:

In cases when it is “boiling” and difficulties are experienced within the nursing community, it is urgent for me to know that anyone knows what the issue revolves around. I have experienced that staying in tough situations with very ill patients can be a “heavy” challenge. Colleagues with the capacity to discuss the situation in a respectful way are therefore a very good thing. In the long run, a lack of support from colleagues would be hard and untenable.

Staying together reduced the sense of vulnerability and influenced nurses’ experiences of a situation. It was stated that in cases when tragic things have happened with a patient, then it has been possible to come through with the help of colleagues.

### Encountering a Relative Absence of Sympathy

The data demonstrate that the content of commonalities among colleagues involved a relative absence of sympathy that gave rise to effort; maintaining indispensable relationships and demonstrating commitment to the community of nursing colleagues became challenging. One nurse explained, “It is rather hard when you have a sense of being rejected or ignored and nobody cares”. Negative verbal or nonverbal responses influenced individual nurses’ outcomes, causing them difficulty in understanding what to do and how to do it when they wanted to look out for their colleagues’ best interests. Encountering a relative absence of sympathy triggered nurses’ thoughts and feelings, including frustration, disappointment and a sense of being lonely or excluded. Particularly when they were tired and in vulnerable situations, nurses reacted strongly to having insufficient support. One nurse said, “Then, I’m thin-skinned and absorb each negative comment without reflection”. Clearly, nurses cannot like all of their colleagues, “I can’t like everyone, and everyone does not like me”.

Nonetheless, one negative colleague can create an uncomfortable atmosphere. One nurse commented that specific colleagues’ behavior could be unpleasant even when a patient’s condition was deteriorating: “Nursing practice is then often performed ‘in the middle of a cauldron’”. The nurse’s expression of being in ‘a cauldron’ referred to nurses blaming each other and the emergence of a strong feeling of dislike and antipathy between nursing colleagues. Another nurse stated, “I’m doing complex and very uncomfortable work, and despite this, I don’t get the help that I need from colleagues when caring for very ill patients”. This involves that nurses stand together with a patient and experience that they need help, but there is no response from colleagues. Notably, such situations could revolve around professional disagreement. Nursing colleagues did not always fulfill their tasks as expected. Colleagues could be perceived as “eye-servants”. An ‘eye-servant’ is a metaphor used to describe an individual who attends to his or her duties only when watched by others. Nursing practice requires collaboration, and colleagues with professional knowledge were described as poor collaborators when they engaged in sabotage. One thing is if colleagues without professional nursing knowledge commit sabotage; however, it is quite another thing if professional nurses actually do this. A nurse said, “It hurts, I have reactions, and yes, sometimes such things contribute to a reaction where it slightly tilts for me”.

In the worst case, encountering a relative absence of sympathy caused resentment and recrimination and a loss of the desire to work. One nurse said, *“*The times I most dread going to work is when there has been a collegial conflict. This is one of the most tiring things that exists; it drains your energy”. Insufficient support within collegial relationships can create such strong conflict that it causes nurses to lose sleep and, in turn, feel like everything is “piling up”. The collection of issues that coalesce in “a heap” can make nurses feel that it might be better to quit the profession. One nurse stated, “Sometimes I would rather wish to be the person who puts stones on the pavement in the city center and then knocks the stones into the earth”. However, the inherent obligation in the practice of nursing requires that nurses find appropriate ways of providing support and relating to each other by demonstrating courtesy even when confronted with ambiguous situations. They had to focus all their energy on putting their own needs to the side and instead working by stretching the resilience needed to continue to work within the community of nursing colleagues.

## Discussion

This study highlights that solidarity is significant regardless of what is specifically shared in the community of nursing colleagues. In other words, nurses’ expression of solidarity manifests in several ways. Given that nurses find themselves dependent on their relationships, they are mutually dependent on each other as colleagues, and their relationships influence their individual outcomes. Observations based on previous research indicate that solidarity is an important quality (Benner, 2000; Løgstrup, 1993). Løgstrup (1993) asserts that solidarity is not only something given in advance but is also related to human initiative and perceived as mutual acknowledgment of a common outcome. In addition, the obligation that arises from the content of commonalities informs what to do or how to do it.

The results demonstrate that solidarity, i.e., commonality, stemmed not only from indispensable relationships characterized by regard and enriching interactions but also from interactions involving a relative absence of sympathy or antipathy. The obligation to continue sharing aspects commonly related to indispensable relationships, i.e., consideration of colleagues’ best interests, thus became more challenging; for example, colleagues could create an uncomfortable atmosphere and behavior could be unpleasant even when a patient’s condition was deteriorating. Such efforts influenced the issues that individual nurses considered. Accountability was not always in accordance with expectations of the important components of nursing care. Other researchers have documented that when the absence of sympathy is pervasive among nurses, defending oneself and asking colleagues for needed assistance becomes difficult (Becher & Visovsky, 2012; Budin et al., 2013; Demir & Rodwell, 2012; Purpora & Blegen, 2015; Rodwell & Demir, 2012; Topa & Moriano, 2013; Vessey et al., 2010). Moreover, an absence of sympathy can make it difficult for nurses to remain in their positions, particularly when they also experience relatively destructive behaviors or violence perpetrated by patients (Beattie et al., 2019; Kristoffersen & Friberg, 2017). In the long run, the consequence can be turnover, which may be the last resort for nurses endeavoring to take care of themselves and avoid health problems (Purpora & Blegen, 2015, Topa & Moriano, 2013; Vessey et al., 2010; Kristoffersen, 2019b). Nevertheless, the obligation to continue sharing aspects commonly related to indispensable relationships is maintained by colleagues’ support for each other, verbal expressions of their appreciation of seeing each other and opportunities to vent unresolved feelings and issues, implying contributions to the experience of being on the same wavelength. Solidarity can be seen as “an exchange of beliefs and desires bonded on mutual feelings of respect and dignity” (Schwartz & Abbott, 2007, p. 185). Prainsack and Buyx (2018) claim that solidarity is “enacted commitments to accept costs to assist others with whom a person or persons recognize similarity in a relevant respect” (p. 588).

Moreover, solidarity is a quality of the community that is related to nurses’ specific situation as professionals. This finding relates to one of the profession’s core competences, i.e., teamwork and collaboration (Institute of Quality and Safety Education for Nurses, 2003), and is in line with what Uslusoy and Alpar (2013) describe as emotional solidarity, i.e., trusting and establishing relationships with colleagues. Collaboration is associated with collegial support, including assistance with job-related challenges (Bjarnadottir, 2011; Elenbecker, 2006; Price, 2001), and with what Benner (2000) describes as respect in relation to professional conduct. Professional conduct for nurses includes an obligation to aid each other in performing nursing practice (Benner et al., 2010; Havig et al., 2013; International Council of Nurses, 2012) and preventing inadequate care (Zuniga et al., 2015). Inadequate care of patients often involves situations in which the suffering of others does not solicit a response of trust, mercy and an openness to listen; however, good nursing practice relies on “embodied capacities to experience” such life manifestations (Benner, 2000, p. 14). Interestingly, in the current study, nurses considered colleagues to be the best qualified to provide support. Their support was often based on embodied professional knowledge; their colleagues understood the difficulties that arose in various situations and frequently remained together in situations when tragic events occurred. This seems consistent with knowledge about the importance of embodied professional knowledge, signifying the integrated and personal qualities of professional knowledge (Benner, 2000; Kristoffersen et al., 2020), and with what Uslusoy and Alpar (2013) describe as the academic dimension of solidarity. Uslusoy and Alpar state that this dimension incorporates the sharing of professional knowledge, methods and skills and thereby contributes to developing the competence of colleagues; such support both develops the competence of colleagues and increases productivity by enabling efficient collaboration.

The study results are not surprising, but they notably highlight that solidarity is experienced as something ambiguous. The content or the obligation that arises from what is common may take varying and opposing forms. Contrasts such as good and bad exist and give rise to a relevant question: is the solidarity among this community of nursing colleagues peripheral to what solidarity generally involves in human relationships? Based on Løgstrup (1993), the study results cannot be understood from like this stance. Løgstrup (1993) states that the content of what is common remains open and can be anything; it is thus not solely good but has to be understood in context. Accordingly, relating solidarity to anything highlights that the study results cannot be understood in line with Uslusoy and Alpar’s (2013) statement. These authors state that solidarity protects nurses from undesirable and destructive behaviors, which is a very different view and indicates that the solidarity of the community of nursing colleagues in this study was peripheral to what solidarity involves. Other researchers support Uslusoy and Alpar. Increased solidarity has been found to be associated with lower levels of incivility, implying that solidarity is perceived as a contribution to the common good, helping others in need, sharing responsibility and apologizing for mistakes (Benner, 2000; Cramm et al., 2013; Itzkovich & Heilbrunn, 2016). However, solidarity, i.e., the obligation that arises from the content of what is common within a context, can include what is perceived as an absence of sympathy, which often occurs when a situation is characterized by insecurity or when something about another person contributes to a perception of a lack of safety or a reaction such as antipathy (Løgstrup, 1991). Therefore, solidarity cannot protect against antipathy, and undesirable actions and behaviors are not understood as an expression of a lack of solidarity. Importantly, the relative absence of sympathy does not necessarily result in damaging the indispensable relationships among the nurses; instead, the nurses often found appropriate ways to relate to each other regardless of the ambiguity of the situation.

## Strengths and Limitations

Credibility, as described by Lincoln and Guba (1985), is enhanced because the data can be considered suitable. The data demonstrated that the daily experiences of nursing practice were relevant for exploring solidarity within a community of nursing colleagues. The participants in the original empirical study had experiences of events within their community of colleagues. The collection of data continued to the point where a sense of closure was attained (Polit & Beck, 2018). Irrelevant data were not included, and the themes covered the data. The study aim was kept in mind throughout the data collection and analysis. To limit the risk of overinterpretation of the data, alternative interpretations in light of the concept of solidarity were considered. Moreover, the principles of methodological support as described by Lindberg et al. (2016) facilitated capturing the information in the data. Posing questions to the text was a useful means of encouraging subsequent analysis. Thus, the analysis was conducted with no predetermined themes. These features indicate that the interpretation of the data was conducted with openness and reflection and can be considered confirmable since the intended focus of the study was addressed (Lincoln & Guba, 1985). Another issue is that the data may be considered limited because of the translation from Norwegian to English, which resulted in the loss of some naturally occurring richness in daily language. No interaction was identified between the researchers’ characteristics and the research questions, approach, method or study results. The research process was sufficiently described, indicating that the data collection and analysis were conducted such that the study results are dependable (Lincoln & Guba, 1985). The results are suggestive at best, implying that they may not be transferable to other contexts since transferability is “an empirical issue” (Lincoln & Guba, 1985, p. 316). Nonetheless, the results might have some relevance to other communities of nursing colleagues (Bjarnadottir, 2011; Elenbecker, 2006; Purpora & Blegen, 2015).

## Implications for Practice

Solidarity can be applied as a background for organizations and leadership to further the understanding of interactions within communities of nursing colleagues. Communities have been shown to influence nurses’ collaborative opportunities to perform high-quality, effective nursing practice (Benner et al., 2010). Nevertheless, colleagues can influence nurses by diminishing the desire to work and remain in the current position (Gilmartin, 2012; Liebermann et al., 2015; Kristoffersen, 2021). Nursing leadership is therefore key. One specific responsibility of nursing leaders is to promote close reflection on the dynamics in interactions among nursing colleagues. This involves considering the qualities of the community of nursing colleagues and attending to the fact that solidarity can shift from something good to something relatively good and vice versa. Another specific responsibility is to promote the acknowledgment of solidarity as a significant quality. This involves investing in adequate strategies and actions that build solidarity by fostering supportive colleagues and counteracting a relative absence of sympathy (Cramm et al., 2013; Prainsack & Buyx, 2018**)**. Alternatively, nurses should be supported when they experience a lack of collegial support, which can directly impact the advancement of nursing practice by improving nurses’ competence and capacity to achieve their professional goals (Bjarnadottir, 2011; Elenbecker, 2006; Uslusoy & Alpar, 2013). This study also leads to a further question of how the aspects of solidarity are connected more explicitly to nursing leadership and which leadership strategies can better prepare nurses to be attuned to their communities. This question should be elaborated upon in the future.

## Conclusion

A sense of community among nursing colleagues seems to rely on solidarity: whatever affects one nurse affects another. The solidarity that arose from the content of commonalities involved maintaining indispensable relationships with nursing colleagues by supporting and aiding them and simultaneously enduring a relative absence of sympathy. Solidarity among the community in this study was not a peripheral concept of the general notion of solidarity, implying that the commonalities within the collegial relationships were ambiguous and could shift from something good to something relatively good and vice versa. Such a shift was evidenced by nurses’ experiences of their community.
